# Stringently Defined Otitis Prone Children Demonstrate Deficient Naturally Induced Mucosal Antibody Response to *Moraxella catarrhalis* Proteins

**DOI:** 10.3389/fimmu.2017.00953

**Published:** 2017-08-11

**Authors:** Dabin Ren, Timothy F. Murphy, Eric R. Lafontaine, Michael E. Pichichero

**Affiliations:** ^1^Rochester General Hospital Research Institute, Rochester, NY, United States; ^2^Clinical and Translational Research Center, Jacobs School of Medicine and Biomedical Sciences, University at Buffalo, The State University of New York, Buffalo, NY, United States; ^3^Department of Infectious Diseases, College of Veterinary Medicine, University of Georgia, Athens, GA, United States

**Keywords:** otitis prone, nasopharyngeal colonization, acute otitis media, immunogenicity, recombinant proteins, antigen, mucosal immune response, carriage

## Abstract

*Moraxella catarrhalis* (*Mcat*) is a prominent mucosal pathogen causing acute otitis media (AOM). We studied *Mcat* nasopharyngeal (NP) colonization, AOM frequency and mucosal antibody responses to four vaccine candidate *Mcat* proteins: outer membrane protein (OMP) CD, oligopeptide permease (Opp) A, hemagglutinin (Hag), and Pilin A clade 2 (PilA2) from stringently defined otitis prone (sOP) children, who experience the greatest burden of disease, compared to non-otitis prone (NOP) children. sOP children had higher NP colonization of *Mcat* (30 vs. 22%, *P* = 0.0003) and *Mcat*-caused AOM rates (49 vs. 24%, *P* < 0.0001) than NOP children. Natural acquisition of mucosal antibodies to *Mcat* proteins OMP CD (IgG, *P* < 0.0001), OppA (IgG, *P* = 0.018), Hag (IgG and IgA, both *P* < 0.0001), and PilA2 (IgA, *P* < 0.0001) was lower in sOP than NOP children. Higher levels of mucosal IgG to Hag (*P* = 0.039) and PilA2 (*P* = 0.0076), and IgA to OMP CD (*P* = 0.010), OppA (*P* = 0.030), and PilA2 (*P* = 0.043) were associated with lower carriage of *Mcat* in NOP but not sOP children. Higher levels of mucosal IgG to OMP CD (*P* = 0.0070) and Hag (*P* = 0.0003), and IgA to Hag (*P* = 0.0067) at asymptomatic colonization than those at onset of AOM were associated with significantly lower rate of *Mcat* NP colonization progressing to AOM in NOP compared to sOP children (3 vs. 26%, *P* < 0.0001). In conclusion, sOP children had a diminished mucosal antibody response to *Mcat* proteins, which was associated with higher frequencies of asymptomatic NP colonization and NP colonization progressing to *Mcat*-caused AOM. Enhancing *Mcat* antigen-specific mucosal immune responses to levels higher than achieved by natural exposure will be necessary to prevent AOM in sOP children.

## Introduction

Acute otitis media (AOM) is the most common infectious disease among children to cause parents to seek medical care for their child and receive antibiotics. *Moraxella catarrhalis* (*Mcat*) has been ranked as the third most common cause of AOM after *Streptococcus pneumoniae* (*Spn*) and non-typeable *Haemophilus influenzae* (NTHi) in children (1). However, the virulence of *Mcat* as an otopathogen may be increasing as evidenced by an increased occurrence of tympanic membrane (TM) rupture caused by the organism (Pichichero, unpublished observation). Our recent studies on the prevalence of otopathogens show that *Mcat* has overtaken *Spn* and NTHi to become the most frequent cause of episodic and recurrent AOM in children (2). Similarly, *Mcat* was recently identified as the most common otopathogen in Finish children (3). AOM often recurs and poses a high burden on the quality of life of children, the health care system and the economy worldwide (4).

There is no licensed vaccine for *Mcat*, and the development of an *Mcat* vaccine is currently moving from animal studies toward clinical trials. A number of antigens have been identified as potential *Mcat* vaccine candidates, of which outer membrane protein (OMP) CD, oligopeptide permease (Opp) A, hemagglutinin (Hag), and Pilin A clade 2 (PilA2) are promising representatives. OMP CD is a porin and adhesin and is highly conserved with exposed epitopes on the bacterial surface (5, 6). OppA is a highly conserved oligopeptide binding protein mediating the uptake of peptides and fitness of the organism in the respiratory tract (7, 8). Hag, also known as *Moraxella* IgD-binding protein (MID), is an adhesin, Hag, and autotransporter containing surface exposed and conserved epitopes (9). PilA2 is a conserved pilin involved in natural genetic transformation, biofilm formation, and adherence of the bacteria to human epithelial cells (10–12).

Otitis prone (OP) defines a health status of children who have recurrent AOM, with at least three episodes in 6 months or four episodes in a 12-month time span (13, 14). To meet the definition of stringently defined otitis prone (sOP), a child must have every episode proven by a tympanocentesis-derived middle ear fluid (MEF) positive culture of an otopathogen while non-otitis prone (NOP) children are those with 0–2 episodes of AOM per year (14, 15). Because sOP children are most vulnerable to AOM, they should be the focused population of vaccination against AOM. Evaluating naturally induced humoral immune responses to *Mcat* proteins may disclose the immunogenicity of these antigens and functional activity of the antibodies elicited by these proteins in the targeted age and most vulnerable population, especially sOP children. We have found that during nasopharyngeal (NP) colonization by *Mcat* all four antigens, OMP CD, OppA, Hag, and PilA2, are immunogenic in both sOP and NOP children (16). The age-dependent increase of naturally induced serum antibody ranked as OppA > Hag5–9 > OMP CD > PilA2 in both sOP and NOP children (Ren et al., unpublished). We also found that sOP children have deficient production of serum antibodies to OMP CD, OppA, Hag, and PilA2 at asymptomatic *Mcat* NP colonization and onset of AOM (Ren et al., unpublished), similar to our findings for protein vaccine candidates of *Spn* (17) and NTHi (18).

*Moraxella catarrhalis* is a mucosal pathogen and mucosal immunity plays an important role in host defense against *Mcat* infections. Here, we studied *Mcat* NP colonization, AOM frequency and mucosal antibody responses to four vaccine candidate *Mcat* proteins, OMP CD, OppA, Hag, and PilA2 in nasal washes from sOP compared to NOP children to identify differences in pathogenicity and mucosal immune responses.

## Materials and Methods

### Subjects and Sampling

The samples collected and analyzed were obtained during a prospective study supported by the National Institute of Deafness and Communication Disorders, as previously described (19, 20). Healthy children without previous episodes of AOM were enrolled at 6 months of age from a middle class, suburban sociodemographic pediatric practice in Rochester, NY, USA during years 2006–2016. For this study, we assessed a total of 628 children followed prospectively until 36 months of age. NP and oropharyngeal cultures were obtained seven times during the study period at 6, 9, 12, 15, 18, 24, and 30–36 months of age. Whenever children experienced an AOM episode, a confirmatory tympanocentesis was performed, and MEF was microbiologically assessed (an essential component to the definition of “stringently defined” AOM since virtually all prior studies have relied on only a clinical diagnosis that is known to be variably accurate). NP, oropharyngeal, and MEF sampling was conducted as previously described (19, 21), and the samples were analyzed to identify bacterial pathogens by using standard culture and PCR assay (19). The study was approved by the Rochester General Hospital Research Subjects Review Boards. Written informed consent was obtained for participation, and all procedures are in accordance with the Declaration of Helsinki.

Nasal wash samples were obtained by instilling 1 ml of sterile phosphate-buffered saline (PBS) into each nare and then aspirating from each nare yielding approximately 2 ml of material for each subject. The nasal wash solution was subsequently centrifuged at 3,000 rpm (1,100 × g) at 4°C for 10 min, and the supernatant was then stored at −80°C until used for quantification of mucosal antibody by using enzyme-linked immunosorbent assay (ELISA).

### Enzyme-Linked Immunosorbent Assay

Recombinant *Mcat* proteins OMP CD, OppA, Hag5–9 (truncated Hag protein), and PilA2 were expressed and purified as previously described (7, 9, 11, 22). Protein-specific antibody concentrations were determined by ELISA using purified recombinant proteins. Three hundred eighty-four well Greiner Microlon plates were coated with 1–3 µg/ml of individual proteins (20 µl/well) in PBS (pH 7.4) and incubated at 37°C for 1 h. After five washes, the plates were blocked with 10% fetal bovine serum in PBS (pH 7.4) at room temperature for 1 h (40 µl/well). After washing, 20 µl of nasal wash twofold serially diluted in a buffer of PBS, 0.5% BSA, with 0.005% Tween was added to each well. Carimune (CSL Behring AG, Bern, Switzerland) for OMP CD IgG assay and Gammagard (Baxter, Deerfield, IL, USA) for OppA, Hag, and PilA2 IgG assays or in-house human sera for IgA assays were used as references and in-house control sera with high and low titers were run on each plate. The plates were incubated at room temperature for 30 min followed by the addition of affinity-purified goat antihuman IgG/IgA antibody conjugated to horseradish peroxidase (Bethyl Laboratories, Montgomery, TX, USA) as a secondary antibody. The reaction products were developed with TMB Microwell Peroxidase Substrate System (KPL, Gaithersburg, MD, USA), stopped by addition of 1.0 M phosphoric acid and read by a Spectramax 340PC plate reader (Molecular Devices, Sunnyvale, CA, USA) using a 450-nm filter.

To provide quantitative results on antibody concentrations, the level of the specific antibody present in the unknown sample was determined by comparing to an internal reference serum (Carimune for OMP CD IgG and Gammagard for OppA IgG, Hag IgG, and PilA2 IgG or in-house human IgA sera). The levels of IgG and IgA in the reference serum were quantitatively measured by using a human IgG or IgA ELISA quantitation kit (Bethyl laboratories). A four-parameter logistic-log function was used to form the reference and sample curves. This ELISA was validated according to the International Council for Harmonization Guidance. The inter-assay coefficient of variation was ≤30% for all antigens and secondary antibody combinations.

### Statistical Analysis

Fisher’s exact test was used to analyze the difference of rates for *Mcat* NP colonization, AOM and NP colonization progressing to AOM. The Mann–Whitney test was used to compare differences in *Mcat* protein-specific IgG or IgA concentrations, normalized by total IgG or IgA concentration in nasal wash, respectively. *P* values of <0.05 were considered significant.

## Results

### Rates of *Mcat* NP Colonization and Percentages of Children with *Mcat*-Caused AOM in sOP and NOP Children

**Figure 1:**
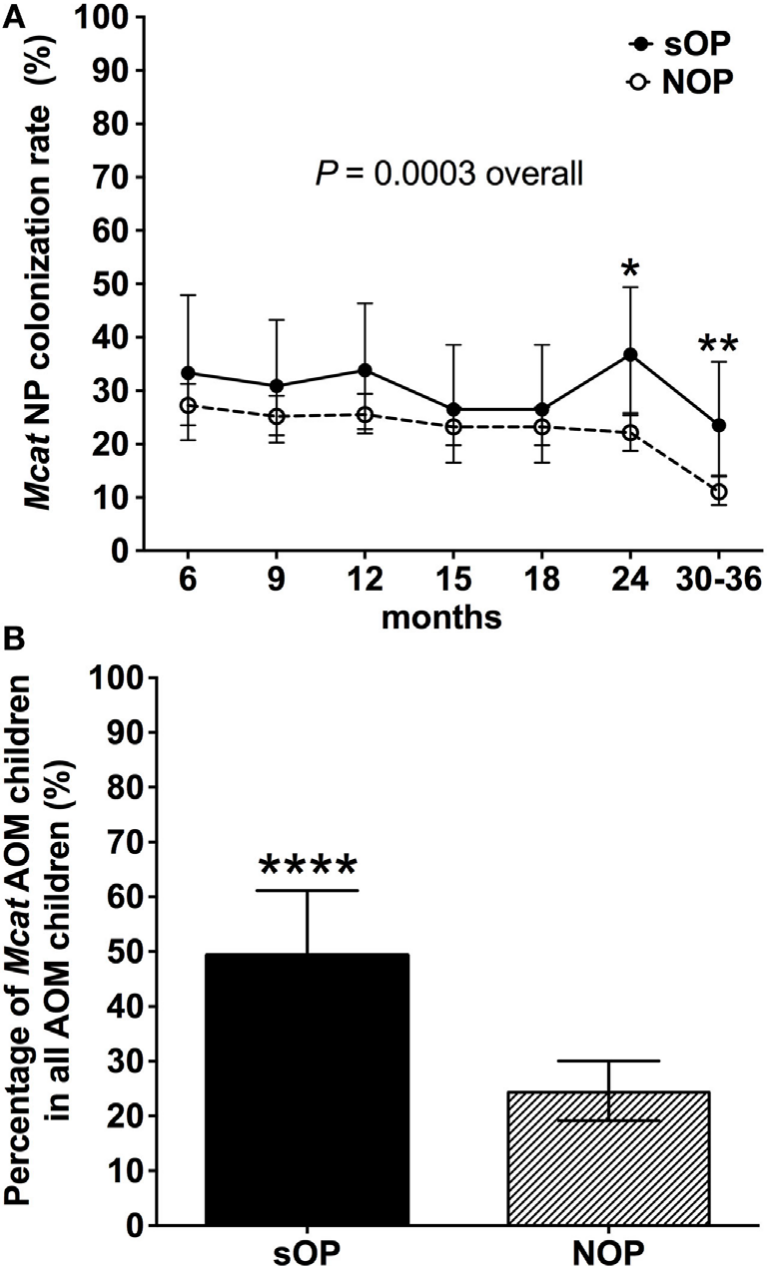
*Moraxella catarrhalis* (*Mcat*) nasopharyngeal (NP) colonization rates and percentages of *Mcat*-caused acute otitis media (AOM) in stringently defined otitis prone (sOP) and non-otitis prone (NOP) children. **(A)** 60 sOP children and 560 NOP children were assessed for *Mcat* NP colonization rates at 6, 9, 12, 15, 18, 21, 24, and 30–36 months old, respectively. **(B)** 75 sOP children and 255 NOP children with AOM history were assessed for the percentage of children with *Mcat*-caused AOM. Data are represented as percentage ± 95% confidence interval. Fisher’s exact test was used to compare the difference of rates or percentages between sOP and NOP children. *P < 0.05, **P < 0.01, and ****P < 0.0001 comparing sOP vs. NOP children.

Nasopharyngeal colonization of otopathogens is the first step in pathogenesis of AOM (23). We analyzed the rates of *Mcat* NP colonization in 68 sOP and 560 NOP children and assessed the percentage of children with *Mcat*-caused AOM in each population (Figure 1). *Mcat* NP colonization rates were consistently higher in sOP vs. NOP children: 33 vs. 27% at age 6 months, 31 vs. 25% at age 9 months, 34 vs. 26% at age 12 months, 26 vs. 23% at age 15 months, 26 vs. 23% at age 18 months, 36 vs. 22% at age 24 months (*P* = 0.010), 23 vs. 11% at age 30–36 months (*P* = 0.006), and overall 30 vs. 22% at age 6–36 months old (*P* = 0.0003, Figure 1A). The relative risk of *Mcat* NP colonization for sOP:NOP children was 1.7 at age 24 months old, 2.1 at age 30–36 months old, and overall 1.3-fold higher for age 6–36 months old.

Thirty seven of 75 (49%) sOP children with AOM had *Mcat*-caused AOM, which is significantly higher than 62 of 255 (24%) NOP children with AOM (*P* < 0.0001, Figure 1B). The relative risk of *Mcat*-caused AOM for sOP:NOP was thus twofold higher.

### Mucosal Antibody Levels Against *Mcat* Proteins in sOP and NOP Children

**Figure 2:**
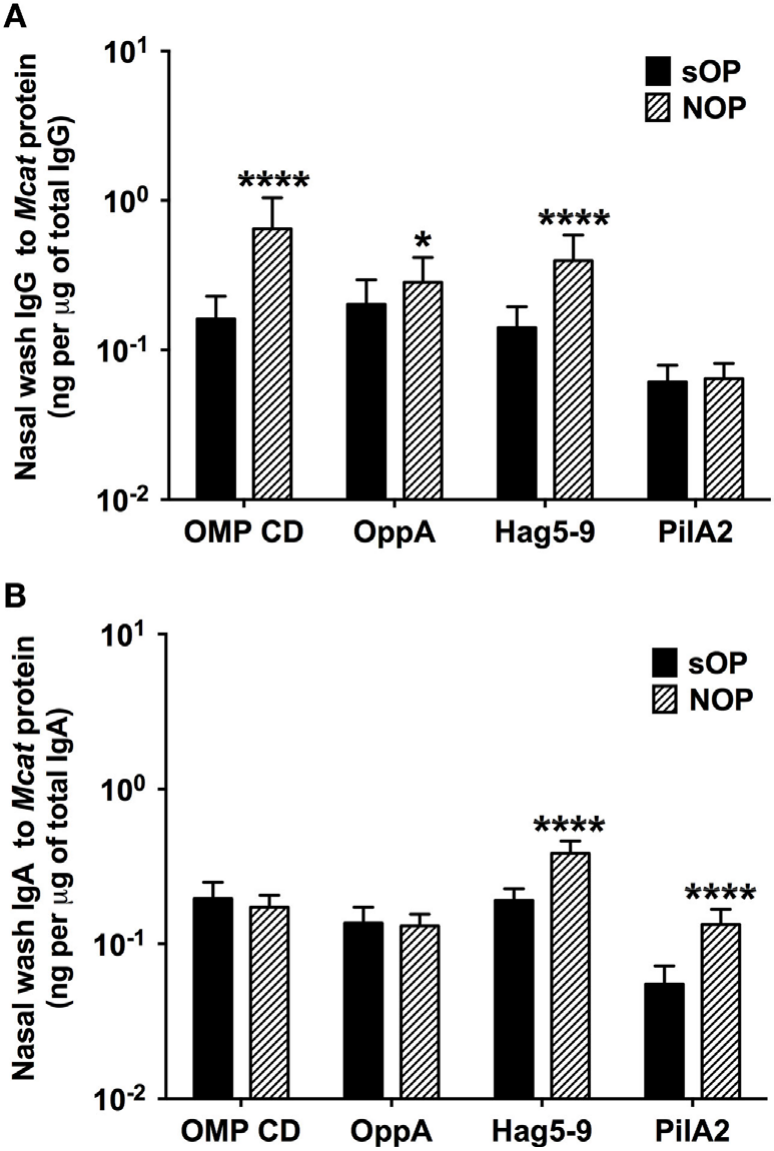
Mucosal antibody levels against *Moraxella catarrhalis* (*Mcat*) proteins in stringently defined otitis prone (sOP) and non-otitis prone (NOP) children. Nasal wash IgG and IgA against *Mcat* proteins outer membrane protein (OMP) CD, oligopeptide permease A (OppA), Hag5–9, and Pilin A clade 2 (PilA2) were detected by using enzyme-linked immunosorbent assay for children age 6–36 months old. *Mcat* protein-specific **(A)** IgG and **(B)** IgA in nasal wash were compared between 238 healthy visits of 59 sOP children and 270 healthy visits of 75 NOP children. IgG and IgA concentrations (ng/ml) were normalized with total IgG and IgA concentrations (μg/ml) in nasal wash, respectively, for comparison. Data are represented as geometric mean ± 95% confidence interval. Mann–Whitney test was used for comparisons. *P < 0.05 and ****P < 0.0001 comparing sOP vs. NOP children.

We analyzed mucosal IgG and IgA antibodies in nasal wash samples from 238 healthy visits of 59 sOP children and 270 healthy visits of 75 NOP children age 6–36 months old. sOP children had significantly lower levels of mucosal IgG to OMP CD (*P* < 0.0001), OppA (*P* = 0.018), and Hag5–9 (*P* < 0.0001) than NOP children (Figure 2A). Similarly, sOP children had significantly lower levels of mucosal IgA antibody to Hag5–9 (*P* < 0.0001) and PilA2 (*P* < 0.0001) than NOP children (Figure 2B). We did not find a significant difference of mucosal IgG and IgA to the four *Mcat* proteins among different age time points 6, 9, 12, 15, 18, 24, and 30–36 months in either sOP or NOP children (*P* > 0.05). Mucosal IgG levels to four proteins could be ranked as OMP CD = OppA = Hag5–9 > PilA2 (*P* < 0.0001) in sOP and NOP children altogether. Mucosal IgA levels to the four proteins could be ranked as Hag5–9 > OMP CD > OppA > PilA2 (*P* < 0.0001) in sOP and NOP children altogether (Figure 2).

### Mucosal Antibodies to *Mcat* Proteins at Healthy Visits with vs. without NP Colonization in sOP and NOP Children

**Figure 3:**
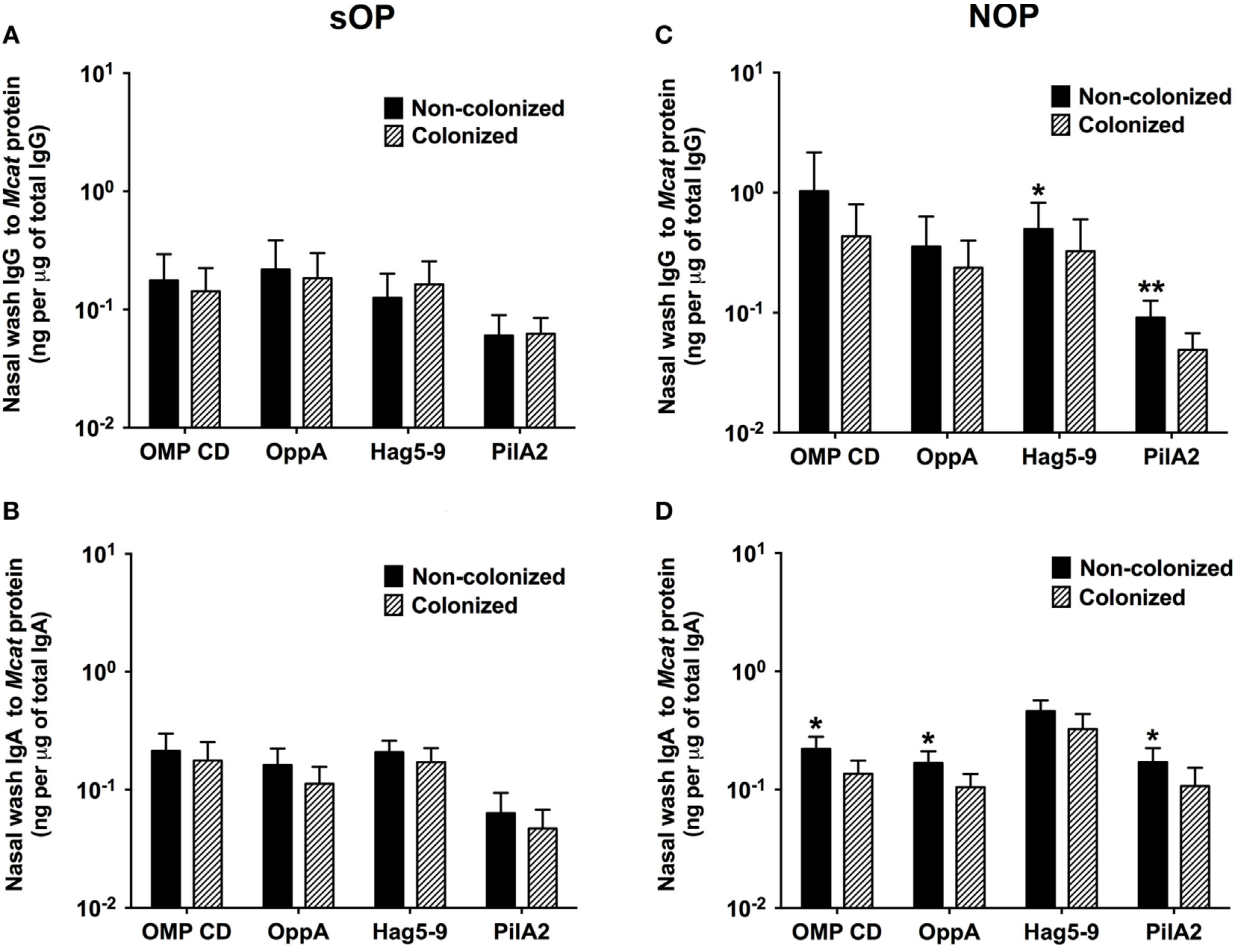
Comparison of mucosal antibodies to *Moraxella catarrhalis* (*Mcat*) proteins between visits with and without *Mcat* nasopharyngeal (NP) colonization in stringently defined otitis prone (sOP) and non-otitis prone (NOP) children. Nasal wash IgG and IgA against *Mcat* proteins outer membrane protein (OMP) CD, oligopeptide permease A (OppA), Hag5–9, and Pilin A clade 2 (PilA2) were detected by using enzyme-linked immunosorbent assay for children age 6–36 months old. **(A,B)** 107 healthy visits with *Mcat* NP colonization positive and 131 healthy visits with *Mcat* NP colonization negative of 59 sOP children were analyzed for nasal wash **(A)** IgG and **(B)** IgA. **(C,D)** 130 healthy visits with *Mcat* NP colonization positive and 140 healthy visits with *Mcat* NP colonization negative of 75 NOP children were analyzed for nasal wash **(C)** IgG and **(D)** IgA. *Mcat* protein-specific IgG and IgA concentrations (ng/ml) were normalized with total IgG and IgA concentrations (μg/ml) in nasal wash, respectively, for comparison. Data are shown as geometric mean ± 95% confidence interval. Mann–Whitney test was used for comparison. *P < 0.05 and **P < 0.01 comparing *Mcat* NP non-colonized vs. colonized visits in the same population of children.

We compared the association of NP colonization status with mucosal antibody responses between sOP and NOP children at age 6–36 months old. We analyzed 107 healthy visits with positive *Mcat* NP colonization and 131 healthy visits with negative *Mcat* NP colonization for 59 sOP children. sOP children did not show a difference of mucosal IgG (Figure 3A) or IgA (Figure 3B) to any of the four protein antigens comparing *Mcat* NP colonization positive and negative visits. We also analyzed 130 healthy visits with *Mcat* NP colonization positive and 140 healthy visits with *Mcat* NP colonization negative for 75 NOP children. In contrast, NOP children without *Mcat* NP colonization had significantly higher mucosal IgG to Hag5–9 (*P* = 0.039) and PilA2 (*P* = 0.0076) than NOP children with *Mcat* NP colonization (Figure 3C). NOP children without *Mcat* NP colonization displayed a significantly higher mucosal IgA to OMP CD (*P* = 0.010), OppA (*P* = 0.030), and PilA2 (*P* = 0.043) than NOP children with *Mcat* NP colonization (Figure 3D).

In addition, mucosal IgG and IgA to *Mcat* proteins were examined for 50 healthy visits of 22 NOP children currently not colonized by *Mcat* but with *Mcat* colonization history and compared to those for 90 healthy visits of 25 NOP children who had never been colonized by *Mcat* (Figure S1 in Supplementary Material). We did not find a significant difference of mucosal antibody levels between these two groups of children (*P* > 0.05, Figure S1 in Supplementary Material). Two out of 22 (9%) NOP children currently not colonized by *Mcat* but with *Mcat* NP colonization history experienced one episode of *Mcat*-caused AOM whereas 3 out of 25 (12%) of the NOP children never colonized by *Mcat* experienced one to two episodes of *Mcat*-caused AOM. There was no difference of *Mcat*-caused AOM rate between the two groups of children (*P* > 0.05). We did not find a difference of *Mcat* protein-specific mucosal antibodies between the children experiencing AOM and those without experiencing AOM in either group of NOP children (*P* > 0.05). Therefore, prior *Mcat* NP colonization did not have a significant effect on mucosal anti-*Mcat* protein antibody levels and *Mcat*-caused AOM frequency in the NOP children without current *Mcat* NP colonization; however, the statistical power to detect differences was limited by the samples size of subjects and colonization/AOM events.

### Mucosal Antibodies to *Mcat* Proteins at Healthy Visits with *Mcat* NP Colonization vs. AOM Visits in sOP and NOP Children

**Figure 4:**
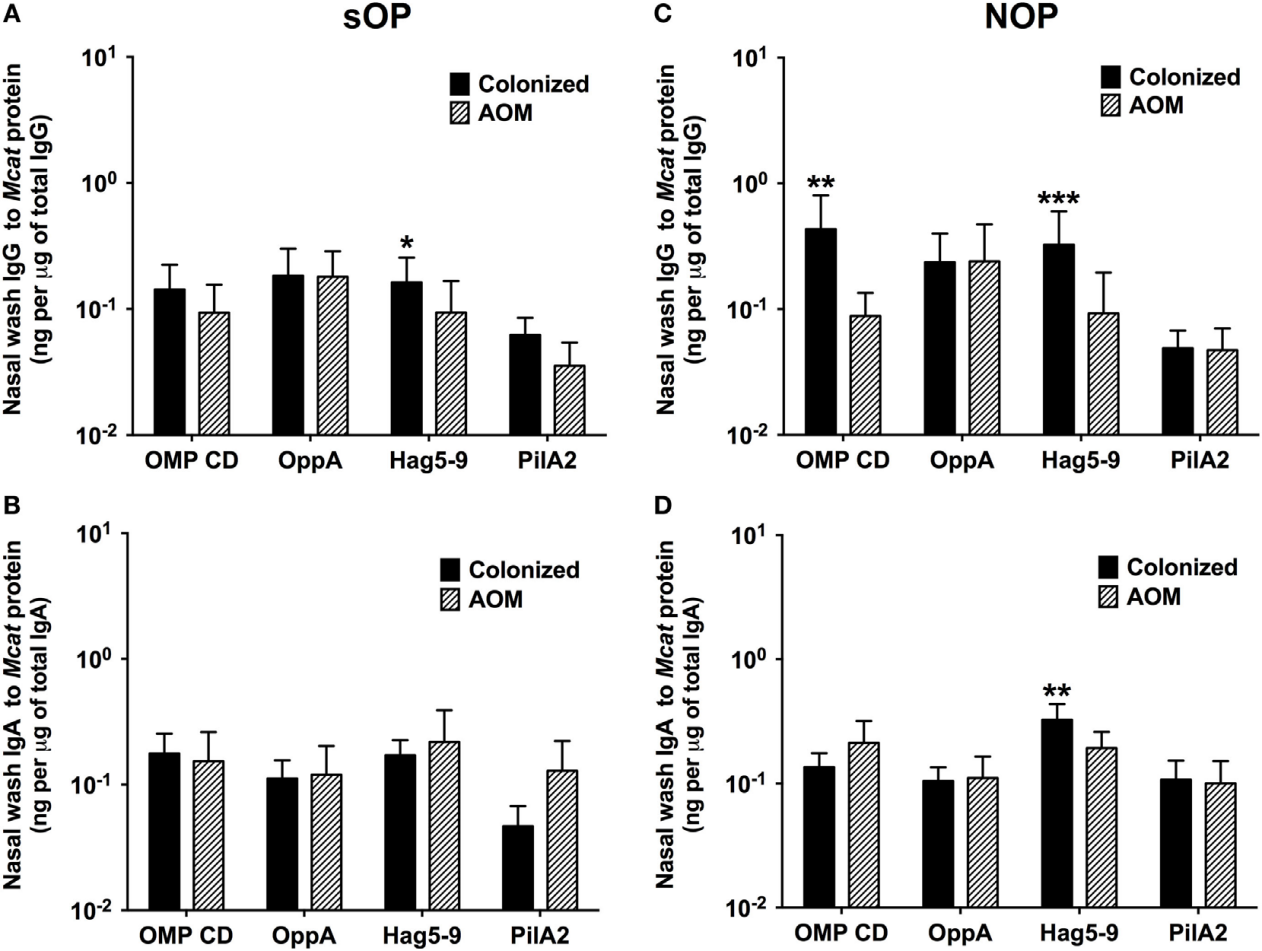
Mucosal antibodies to *Moraxella catarrhalis* (*Mcat*) proteins at healthy visits with *Mcat* nasopharyngeal (NP) colonization vs. acute otitis media (AOM) visits in stringently defined otitis prone (sOP) and non-otitis prone (NOP) children. Nasal wash IgG and IgA against *Mcat* proteins outer membrane protein (OMP) CD, oligopeptide permease A (OppA), Hag5–9, and Pilin A clade 2 (PilA2) were detected by using enzyme-linked immunosorbent assay for children age 6–36 months old. **(A,B)** 107 *Mcat* NP colonization visits of 47 sOP children and 42 *Mcat*-caused AOM visits of 31 sOP children were analyzed for nasal wash **(A)** IgG and **(B)** IgA. **(C,D)** 130 *Mcat* NP colonization visits of 61 NOP children and 43 *Mcat*-caused AOM visits of 37 NOP children were analyzed for nasal wash **(C)** IgG and **(D)** IgA. *Mcat* protein-specific IgG and IgA concentrations (ng/ml) were normalized with total IgG and IgA concentrations (μg/ml) in nasal wash, respectively, for comparison. Data are shown as geometric mean ± 95% confidence interval. Mann–Whitney test was used for comparison. *P < 0.05, **P < 0.01 and ***P < 0.001 comparing *Mcat* NP colonized vs. AOM visits in the same population of children.

**Figure 5:**
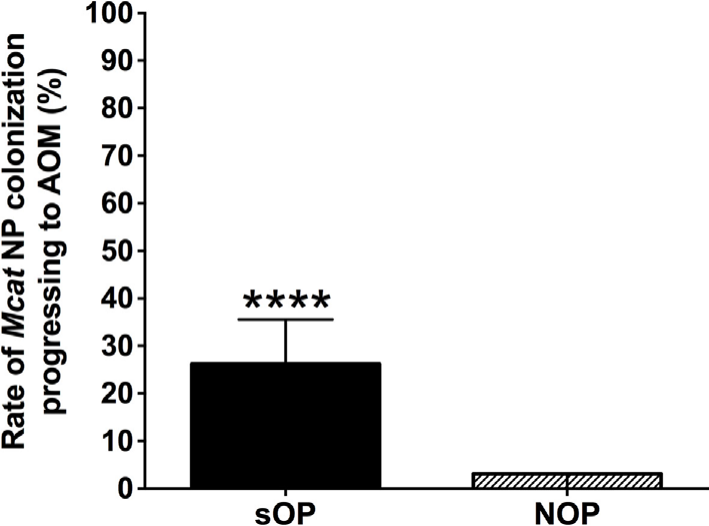
Rates of *Moraxella catarrhalis* (*Mcat*) nasopharyngeal (NP) colonization progressing to acute otitis media (AOM) in stringently defined otitis prone (sOP) and non-otitis prone (NOP) children. Rates of *Mcat* NP colonization progressing to *Mcat*-caused AOM were assessed for 47 sOP and 61 NOP children age 6–36 months old. Data are represented as percentage ± 95% confidence interval (CI). Ninety five percent CI (error bar) for NOP children cannot be calculated by Fisher’s exact test due to only four (less than five) episodes of *Mcat* NP colonization progressing to AOM in NOP children. Fisher’s exact test was used to compare the difference in the rates between sOP and NOP children. ****P < 0.0001 comparing sOP vs. NOP children.

*Moraxella catarrhalis* NP colonization is an immunizing event producing systemic and mucosal antibodies which may confer protection against subsequent NP colonization and AOM infections. We compared the mucosal antibodies at asymptomatic colonization by *Mcat* vs. onset of *Mcat*-caused AOM to analyze the association of natural antibody induction with protection against AOM in both sOP and NOP children. Mucosal IgG and IgA were analyzed for 107 and 130 *Mcat* NP colonization visits of 47 sOP and 61 NOP children, respectively, and 42 and 43 *Mcat*-caused AOM visits of 31 sOP and 37 NOP children, respectively. sOP children did not show a higher mucosal IgG (Figure 4A) or IgA (Figure 4B) to any of the four *Mcat* proteins at *Mcat* NP colonization compared to those at onset of AOM except a higher mucosal IgG to Hag5–9 upon *Mcat* NP colonization than that at onset of AOM (*P* = 0.015, Figure 4A). In contrast, NOP children displayed higher mucosal IgG to OMP CD (*P* = 0.0070, Figure 4C) and Hag5–9 (*P* = 0.0003, Figure 4C) and IgA to Hag5–9 (*P* = 0.0067, Figure 4D) at *Mcat* NP colonization than that at onset of AOM. There was a significantly higher rate of *Mcat* NP colonization progressing to AOM in sOP children (28 in total of 107 *Mcat* NP colonization positive visits, 26%) than that in NOP children (4 in total of 130 *Mcat* NP colonization positive visits, 3%) (*P* < 0.0001, Figure 5). The relative risk of asymptomatic colonization progressing to AOM for sOP was 8.5-fold higher than NOP children.

## Discussion

Otitis prone is a health condition of frequent recurrence of AOM in children, usually younger than 5 years old. In this study, we found that sOP children displayed more frequent AOMs and asymptomatic NP colonizations caused by *Mcat* than NOP children, which is consistent with our prior observations on *Mcat* as well as otopathogens *Spn* and NTHi (24). Our previous study of children enrolled during years 2006–2011 showed that sOP children had a higher *Mcat* NP colonization rate than NOP children at age 6–12 months old (24). However, when we included more subjects enrolled during years 2011–2016, sOP children exhibited higher *Mcat* NP colonization rates than NOP children at a later age phase of 24–36 months old, which suggests that *Mcat* NP colonization trends to be more prevalent in older sOP children during more recent years. Indeed, *Mcat* has been considered a less virulent bacterium than *Spn* or NTHi and has not been associated with TM rupture or severe illness in young children (25). However, during our 10-year (2006–2016) prospective, longitudinal clinical, and translational studies of AOM, only since 2013 have we observed cases of severe AOM symptoms and TM rupture caused by *Mcat* (Pichichero et al., unpublished observations), suggesting that the virulence of *Mcat* as an otopathogen may be increasing. *Mcat* causing TM rupture was first described in Israel in 2009 (25) and in Italy in 2017 (26), suggesting the emergence of more virulent *Mcat* is a potentially significant problem of global proportions. Also, *Mcat* NP colonization rates have significantly increased since the introduction of pneumococcal glycoconjugate vaccines in children (27). The pattern of *Mcat* NP colonization increase in our study may be due to the higher prevalence of emerging virulent *Mcat* strains in older sOP children age 24–36 months.

Studies have shown that AOM has a multifactorial etiology (4). The mechanism of OP has been attributed to many pathogenic factors, such as preceding upper respiratory infection, immature Eustachian tube, social and environmental risk factors (day care environment, parent smoking, multiple siblings at home, etc.) (4), and importantly the delayed maturation of the child’s immune systems as identified by our group (15). In this study, we observed decreased mucosal IgG and IgA responses to all four *Mcat* proteins in sOP children at healthy visits, which is in concert with our prior observations on diminished serum and/or mucosal antibody responses to *Spn* (17, 24), NTHi (18), and *Mcat* (Ren et al., unpublished) protein antigens in this population. In accordance with our prior findings on serum antibody responses (16), high mucosal antibodies correlated with low *Mcat* NP carriage, suggesting protection by naturally acquired *Mcat* protein-specific mucosal antibodies in NOP but not sOP children. Verhaegh and coworkers had similar findings. They showed serum IgG levels to MID962-1200 were higher in 2-year-old children without *Mcat* NP colonization compared to children with *Mcat* NP colonization (28).

Age of the child and preexisting antibody levels are important covariates in predicting an antibody response to NP colonization. Our prior studies showed varied serum IgG responses during NP colonization of *Spn* and NTHi vs. non-NP colonization in children age 6–30 months old (19, 20, 24). *Spn* and NTHi also display differences from *Mcat* in producing acute responses to AOM (29). Thus, different pathogens may evoke different patterns of antibody responses in relation to NP colonization in young children, reinforcing the need for systematic study of each organism in different hosts. However, consistent with our prior observations on antibody responses to *Spn* and NTHi, sOP children tend to produce lower mucosal antibody responses to natural exposure to *Mcat* proteins than NOP children suggestive of a more neonatal-like, delayed immune maturation response.

Mucosal antibodies to OMP CD (22, 30) and Hag (9, 31) have been detected in healthy adults and in those with chronic obstructive pulmonary disease. Mucosal antibodies to Hag have also been observed in young children (32). The current results show that all four *Mcat* protein antigens are immunogenic with regard to mucosal antibodies in young children. The mucosal IgG levels to four *Mcat* proteins could be ranked as OMP CD = OppA = Hag5–9 > PilA2 while mucosal IgA levels to four *Mcat* proteins could be ranked as Hag5–9 > OMP CD > OppA > PilA2 in both sOP and NOP children. The current mucosal data support our prior serum data that OMP CD, OppA, and Hag5–9 exhibit promising immunogenicity for *Mcat* vaccine candidates (16).

Nasopharyngeal colonization of pathogens induces naturally acquired serum and mucosal antibodies, which may confer host defense against infections. The current mucosal results are consistent with our prior serum data that high antibodies to *Mcat* proteins are associated with reduced *Mcat* carriage, suggesting a protective effect of naturally induced antibodies against NP colonization. The observation of reduced mucosal antibodies in the current study is in line with our prior data of decreased serum antibodies to *Mcat* proteins in sOP children during asymptomatic colonization, suggesting that sOP children had a deficient immune responses to *Mcat*. Stenfors and Raisanen found that there are less secretory IgA (sIgA)-coated bacteria in the nasopharynx of OP children, which may be responsible for the otitis-prone condition (33). Our results of lower naturally acquired mucosal *Mcat*-specific IgA support Stenfors’ findings in OP children. Impaired mucosal IgA production could lead to less inhibition of *Mcat* adherence to NP epithelial cells and higher NP colonization in sOP children, which was observed in both the current and prior studies (24, 34).

High frequency of NP colonization of otopathogens *Spn*, NTHi, and *Mcat* is associated with increased development of AOM (35). Among the three otopathogens, early colonization with *Mcat* is associated with the greatest risk of AOM and otitis media with effusion (35). Our results had consistent findings for sOP children of lower production of mucosal IgG and IgA to *Mcat* proteins upon NP colonization and a higher rate of progressing to *Mcat*-caused AOM. On the other hand, NOP children had higher mucosal antibodies to *Mcat* proteins and lower rates of NP colonization progressing to AOM (>8 times lower than sOP children). Mucosal immunity is the frontline of host defense against bacterial infections. Enhanced bactericidal effect of IgG and coating of bacteria with sIgA to inhibit bacterial adherence to respiratory epithelia may account for the protection against AOM observed in this study.

In summary, sOP children had higher NP colonization of *Mcat, Mcat*-caused AOM rates and a higher percentage of *Mcat* NP colonization progressing to *Mcat*-caused AOM than NOP children. These pathogenesis events and outcomes correlate with impaired mucosal antibody responses to *Mcat* protein antigens. Mucosal vaccination with immunogenic *Mcat* antigens and developing mucosal adjuvants should be pursued for preventing AOM caused by *Mcat*, with special attention to the sOP child.

## Author Contributions

DR and MP conceived and designed the study. DR, MP, TM, and EL provided materials. DR performed experiments and statistical analysis. DR and MP wrote the manuscript. All the authors have approved the final manuscript.

## Conflict of Interest Statement

TM has patents for vaccines for *M. catarrhalis*. No other potential conflicts of interest were disclosed.

## Supplementary Material

Figure S1 Comparison of mucosal antibodies to *Moraxella catarrhalis* (*Mcat*) proteins between visits with and without *Mcat* nasopharyngeal (NP) colonization history in non-otitis prone (NOP) children without current *Mcat* NP colonization. Nasal wash IgG and IgA against *Mcat* proteins outer membrane protein (OMP) CD, oligopeptide permease A (OppA), Hag5–9, and Pilin A clade 2 (PilA2) were detected by using enzyme-linked immunosorbent assay for children age 6–36 months old. *Mcat* protein-specific **(A)** IgG and **(B)** IgA in nasal wash were compared between 50 healthy visits of 22 NOP children currently not colonized by *Mcat* but with *Mcat* colonization history and 90 healthy visits of 25 NOP children never colonized by *Mcat*. IgG and IgA concentrations (ng/ml) were normalized with total IgG and IgA concentrations (μg/ml) in nasal wash, respectively, for comparison. Data are represented as geometric mean ± 95% confidence interval. Mann–Whitney test was used for comparisons.

## Abbreviations

*Mcat, Moraxella catarrhalis*; AOM, acute otitis media; OP, otitis prone; sOP, stringently defined otitis prone; NOP, non-otitis prone; NP, nasopharyngeal; OMP, outer membrane protein; *Spn, Streptococcus pneumoniae*; NTHi, non-typeable *Haemophilus influenzae*; OppA, oligopeptide permease A; MID, *Moraxella* IgD-binding protein; Hag, hemagglutinin; TM, tympanic membrane; Msp, *Moraxella* surface protein; PilA2, Pilin A clade 2; MEF, middle ear fluid; ICH, International Council for Harmonization; GM, geometric mean; CI, confidence interval; OME, otitis media with effusion; URI, upper respiratory infection; sIgA, secretory IgA.
