# Supplementary figures and images for: Stringently Defined Otitis Prone Children Demonstrate Deficient Naturally Induced Mucosal Antibody Response to Moraxella catarrhalis Proteins
Source: Front Immunol. 2017 Aug 11;8:953. doi: 10.3389/fimmu.2017.00953 (PMC5554491; doi:10.3389/fimmu.2017.00953)

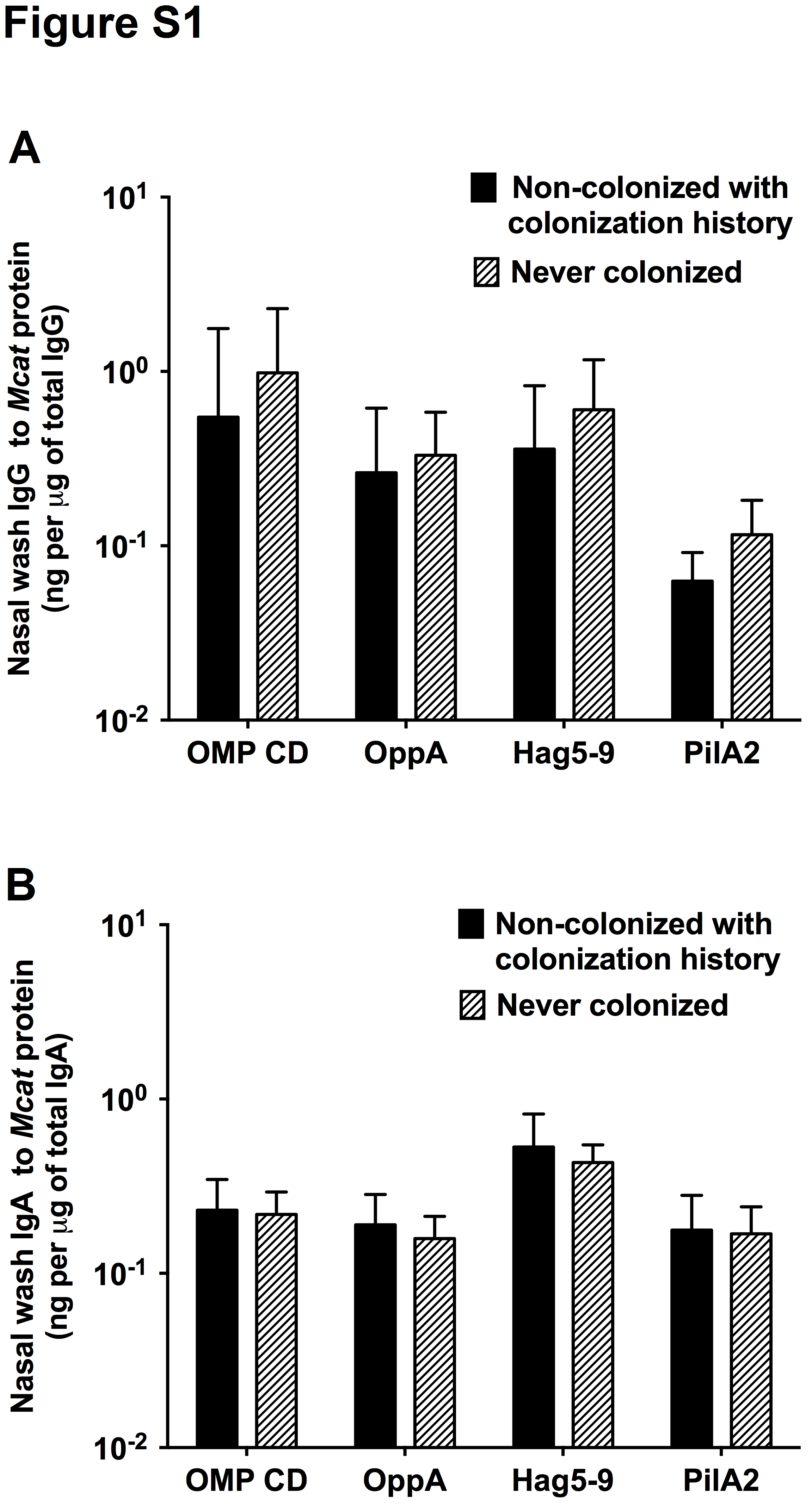

Supplement: Figure S1 — Comparison of mucosal antibodies to Moraxella catarrhalis (Mcat) proteins between visits with and without Mcat nasopharyngeal (NP) colonization history in non-otitis prone (NOP) children without current Mcat NP colonization. Nasal wash IgG and IgA against Mcat proteins outer membrane protein (OMP) CD, oligopeptide permease A (OppA), Hag5–9, and Pilin A clade 2 (PilA2) were detected by using enzyme-linked immunosorbent assay for children age 6–36 months old. Mcat protein-specific (A) IgG and (B) IgA in nasal wash were compared between 50 healthy visits of 22 NOP children currently not colonized by Mcat but with Mcat colonization history and 90 healthy visits of 25 NOP children never colonized by Mcat. IgG and IgA concentrations (ng/ml) were normalized with total IgG and IgA concentrations (μg/ml) in nasal wash, respectively, for comparison. Data are represented as geometric mean ± 95% confidence interval. Mann–Whitney test was used for comparisons. [file image_1.tif]
